# Sex differences in left ventricular remodelling, myocardial fibrosis and mortality after aortic valve replacement

**DOI:** 10.1136/heartjnl-2019-314987

**Published:** 2019-08-29

**Authors:** Anvesha Singh, Tarique Al Musa, Thomas A Treibel, Vassiliou S Vassiliou, Gabriella Captur, Calvin Chin, Laura E Dobson, Silvia Pica, Margaret Loudon, Tamir Malley, Marzia Rigolli, James Robert John Foley, Petra Bijsterveld, Graham R Law, Marc Richard Dweck, Saul G Myerson, Sanjay K Prasad, James C Moon, John P Greenwood, Gerry P McCann

**Affiliations:** 1 Cardiovascular Sciences, University of Leicester and the NIHR Leicester Biomedical Research Centre, Glenfield Hospital, Leicester, UK; 2 Cardiovascular Sciences, Multidisciplinary Cardiovascular Research Centre and The Division of Biomedical Imaging, Leeds Institute for Cardiovascular and Metabolic Medicine, University of Leeds, Leeds, UK; 3 Cardiovascular Sciences, Barts Health NHS Trust and University College London, London, UK; 4 Cardiovascular Sciences, Imperial College London, Royal Brompton Hospital, London, UK; 5 University of East Anglia and Norfolk and Norwich University Hospitals, Norwich, Norfolk, United Kingdom; 6 Cardiovascular Medicine, National Heart Center Singapore, Singapore, Singapore; 7 Cardiovascular Sciences, University of Oxford Centre for Clinical Magnetic Resonance Research, Oxford, UK; 8 Medical Statistics, School of Health and Social Care, University of Lincoln and Multidisciplinary Cardiovascular Research Centre and The Division of Biomedical Imaging, Leeds Institute for Cardiovascular and Metabolic Medicine, University of Leeds, Lincoln and Leeds, UK; 9 Cardiovascular Sciences, Centre for Cardiovascular Science, University of Edinburgh, Edinburgh, UK; 10 Cardiology, Royal Brompton Hospital, London, UK

**Keywords:** aortic stenosis, AVR, CMR

## Abstract

**Objectives:**

To investigate sex differences in left ventricular remodelling and outcome in patients undergoing surgical or transcatheter aortic valve replacement (SAVR/TAVR).

**Methods:**

In this multicentre, observational, outcome study with imaging core-lab analysis, patients with severe aortic stenosis (AS) listed for intervention at one of six UK centres were prospectively recruited and underwent cardiovascular magnetic resonance imaging. The primary endpoint was all-cause mortality and secondary endpoint was cardiovascular mortality.

**Results:**

674 patients (425 men, 249 women, age 75±14 years) were included: 399 SAVR, 275 TAVR. Women were older, had higher surgical risk scores and underwent TAVR more frequently (53% vs 33.6%, p<0.001). More men had bicuspid aortic valves (BAVs) (26.7% vs 14.9%, p<0.001) and demonstrated more advanced remodelling than women. During a median follow-up of 3.6 years, 145 (21.5%) patients died, with no significant sex difference in all-cause mortality (23.3% vs 20.5%, p=0.114), but higher cardiovascular mortality in women (13.7% vs 8.5%, p=0.012). There were no significant sex-related differences in outcome in the SAVR or TAVR subgroups, or after excluding those with BAV. Factors independently associated with all-cause mortality were age, left ventricular ejection fraction (LVEF), BAV (better) and myocardial fibrosis detected with late gadolinium enhancement (LGE) in men, and age, LVEF and LGE in women. Age and LGE were independently associated with cardiovascular mortality in both sexes.

**Conclusions:**

Men demonstrate more advanced remodelling in response to a similar severity of AS. The higher cardiovascular mortality observed in women following AVR is accounted for by women having less BAV and higher risk scores resulting in more TAVR. LGE is associated with a worse prognosis in both sexes.

## Introduction

Male and female patients remodel differently in response to pressure overload/ischaemia induced by aortic stenosis (AS). Remodelling is defined as a change in shape, structure or function of the heart. While echocardiographic studies suggest more concentric remodelling in women,^1 2^ recent cardiovascular magnetic resonance (CMR) imaging studies have confirmed higher left ventricular (LV) volumes, mass index, mass/volume, lower EF and more late gadolinium enhancement (LGE), a marker of focal fibrosis, in men.^3–5^ LGE is associated with adverse prognosis following aortic valve replacement (AVR).^6–8^

Despite more advanced remodelling in men, women appear to have worse outcomes following surgical AVR (SAVR)^9–11^ in some studies but not others.^12 13^ Recent studies using transcatheter AVR (TAVR) have demonstrated a survival benefit in women.^14 15^ Female sex has been found to be an independent predictor of symptom onset in AS^16^ and a predictor of cardiovascular mortality in our recently published multicentre study.^17^ The aim of this study was to investigate the apparent discrepancy between remodelling and outcome between sexes in patients undergoing SAVR/TAVR.

## Methods

### Study design

In this multicentre, longitudinal, observational outcome study conducted in the UK,^17^ patients with severe AS listed for intervention at one of six cardiothoracic surgical units were prospectively recruited. Patients>18 years of age with severe AS (one of the following: aortic valve area (AVA)<1 cm^2^, peak pressure gradient >64 mmHg, mean pressure gradient (MPG) >40 mmHg) who had undergone CMR for research purposes were included. The primary endpoint was all-cause mortality. The secondary endpoint was cardiovascular disease-related mortality, as defined by diagnosis on the UK death certificate. Data were collected on baseline characteristics (demographics, medical and drug history), surgical risk scores (STS V.2.73 and EuroSCORE II) and aortic valve gradients and area from transthoracic echocardiography, at the time of CMR.

### CMR acquisition

CMR was performed on 1.5/3.0 T scanners using standardised protocols including cine imaging for ventricular volumes and function, phase-contrast velocity-encoded imaging for valve haemodynamics and LGE imaging for myocardial scar assessment.^7 18^ All participating centres have previously published single-centre studies in AS, with image quality and CMR pulse sequence parameters.^16 18–21^

### Image analysis and data management

The details of data management and image analysis have been published.^17^ Anonymised data were collected and managed using Research Electronic Data Capture (REDCap) software.^22^ All deaths were identified through the UK National Health Service National Spine Database. Cardiovascular mortality was established in all deceased from the death certificates and adjudicated by two blinded readers (PB and JPG). All CMR scans were re-analysed in core-lab fashion,^17^ with each centre reporting a single component for all patients, after training and reproducibility assessment, and using standardised operating procedures, on CVI42 software (Circle Calgary, Canada). The full-width-half-maximum technique was used to quantify LGE.

### Statistical analysis

Normality was assessed using the Shapiro-Wilk test, histograms and Q-Q plots using SPSS V.24.0 software (Statistical Package for the Social Sciences, Chicago, Illinois, USA). For continuous data, mean±SD for normally distributed data and median(IQR) for non-parametric data are presented. Categorical variables are expressed as counts and percentage. Data between the sexes were compared using independent t-test or Mann-Whitney U test. The Χ^2^ test was used for categorical variables. P values <0.05 were considered statistically significant. Univariate associates of outcomes were determined using Cox proportional hazards models, with the inclusion of sex-interaction variable into the model. Variables for the multivariable models were selected based on statistical significance (p<0.10) and clinical relevance, while avoiding co-linear variables (LV volumes were not included in addition to left ventricular ejection fraction (LVEF), as they are used in its calculation). As Society of Thoracic Surgeons (STS) score incorporates most clinical and demographic variables, this was not included in the initial model, but the added effect of LGE was tested in separate models. Survival was evaluated using the Kaplan-Meier method and compared between sexes using the log-rank test. The index date was the date of CMR. HRs were expressed as mean±95% CI.

## Results

### Baseline characteristic

**Figure 1:**
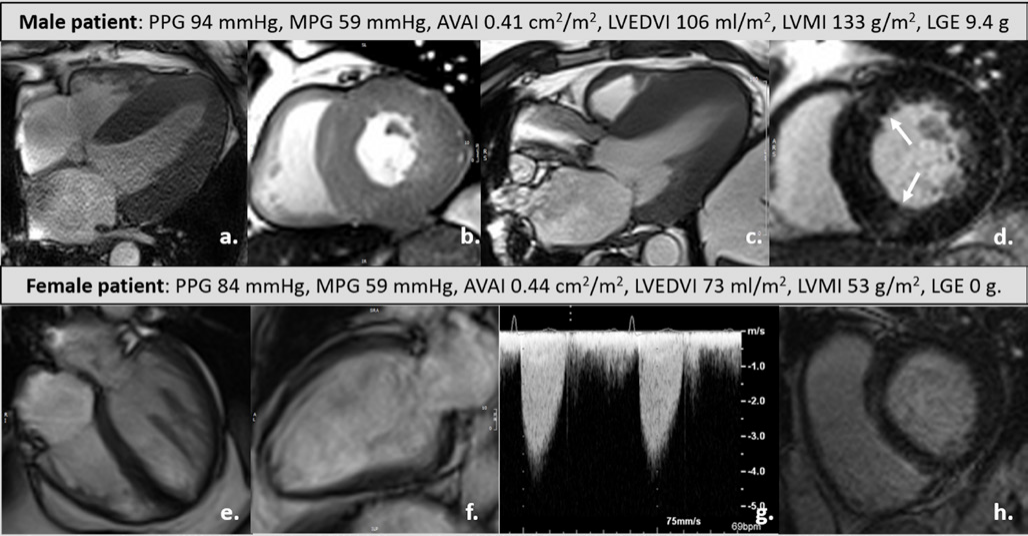
An example of a male (top row) and female (bottom row) patient with similar degree of aortic stenosis, showing cine stills (a-c, e, f), LGE imaging (d, h) and Continuous Wave Doppler through the aortic valve on echocardiography (g). AVAI, aortic valve area index (to BSA); LGE, late-gadolinium enhancement; LVEDVI, left ventricle end-diastolic volume index; LVMI, left ventricle mass index; MPG, mean pressure gradient; PPG, peak pressure gradient.

From 703 patients who underwent CMR, 29 were managed medically, and were excluded from further analysis (15 men and 14 women). This left 674 patients, 425 men and 249 women: mean age 75±14 years, AVA index 0.38±0.14 cm^2^/m^2^, MPG of 46±18 mmHg. Female patients were older, with higher STS and EuroSCORES (table 1). The prevalence of coronary artery disease and bicuspid aortic valve (BAV) was higher in men. AS severity was similar (no statistically significant difference in trans-thoracic echocardiogram (TTE)-measured pressure gradients and corrected AVA). Men demonstrated higher body surface area-corrected ventricular volumes, LV mass and mass/volume; marginally lower EF and a greater prevalence and amount of LGE (figure 1). A greater proportion of women underwent TAVR (53% vs 33.6%, p<0.001). There was no statistically significant difference in age or AS severity of the men and women in either SAVR-only or TAVR-only subgroup.

**Table 1:** Baseline characteristics

Variable	Male (n=425)	Female (n=249)	P value
Age (years)	71.8±10.5	74.9±10.7	**<** **0.001**
BMI (kg/m^2^)	27.8±4.6	27.2±5.8	0.198
SBP (mmHg)	133.9±19.4	136.9±22.1	0.107
Hypertension (n (%))	228 (53.6)	130 (52.2)	0.718
AF (n (%))	47 (11.1)	37 (14.9)	0.149
Diabetes (n (%))	98 (23.1)	48 (19.3)	0.250
Known CAD (n(%))	140 (32.9)	46 (18.5)	**<** **0.001**
Previous PCI (n(%))	31 (7.7)	26 (10.6)	0.203
Previous CABG (n(%))	46 (11.4)	12 (4.9)	**0.005**
ACEI/ARB (n (%))	177 (43.5)	85 (36.8)	0.099
Beta-blocker (n (%))	148 (35.0)	92 (36.9)	0.609
Statin (n (%))	266 (63.8)	140 (57.1)	0.090
BAV (n(%))	112 (26.5)	37 (14.9)	**<** **0.001**
STS mortality score (%)	1.56 [0.98, 2.55)	2.30 [1.32, 4.16)	**<** **0.001**
EuroSCORE II (%)	1.45 [0.91, 3.12)	2.12 [1.32, 4.16)	**<** **0.001**
NYHA class			
I	60 (15.6)	21 (9.3)	**0.020**
II	170 (44.3)	88 (39.1)	
III	143 (37.2)	105 (46.7)	
IV	11 (2.9)	11 (4.9)	
Echocardiographic data			
MPG (mmHg)	48.1±16.4	49.2±14.6	0.451
PPG (mmHg)	80.9±24.9	83.9±23.1	0.157
AVA (cm^2^)	0.76±0.23	0.66±0.23	**<** **0.001**
AVAI (cm^2^/m^2^)	0.38±0.11	0.38±0.13	0.753
Baseline cardiovascular magnetic resonance data			
LVEDVI (mL/m^2^)	87.2±26.5	79.2±22.1	**<** **0.001**
LVESVI (mL/m^2^)	39.2±24.6	33.7±20.1	**0.002**
LVSVI (mL/m^2^)	48.0±12.1	45.5±10.2	**0.006**
LVEF (%)	60.0 [51.0, 67.0)	62.0 [53.0, 69.0)	**0.024**
LVMI (g/m^2^)	88.6±24.5	74.5±21.6	**<** **0.001**
LV mass/volume	1.06±0.29	0.97±0.25	**<** **0.001**
RVEDVI (mL/m^2^)	72.1±16.8	66.1±16.6	**<** **0.001**
RVEF (%)	64.0 [57.0, 70.0)	65.0 [59.0, 73.0)	**0.025**
LAVI (mL/m^2^)	55.6±21.1	58.3±21.9	0.127
LGE present (n(%))	248 (62.6)	93 (42.9)	**<** **0.001**
Non-infarct pattern (n(%))	157 (39.6)	65 (30.0)	**0.017**
Infarct pattern (n(%))	91 (23.0)	28 (12.9)	**0.003**
LGE mass (g)	1.90 [0.00, 6.51)	0.00 [0.00, 2.40)	**<** **0.001**
LGE (%LV mass)	1.12 [0.00, 3.60)	0.00 [0.00, 1.63)	**<** **0.001**
Intervention type			
SAVR (n(%))	282 (66.4)	117 (47.0)	**<** **0.001**
TAVR (n(%))	143 (33.6)	132 (53.0)	**<** **0.001**

P values using independent t-test/Mann-Whitney U test/Χ^2^ test as appropriate. p<0.05 shown in Bold.

ACEI, angiotensin-converting enzyme inhibitor; AF, atrial fibrillation; ARB, angiotensin II receptor blocker; AVA, aorticvalve area; AVAI, aortic valve area index (to BSA); BAV, bicuspidaortic valves; BMI, body mass index; BSA, body surface area; CABG, coronary artery bypass graft; CAD, coronary artery disease; LAVI, left atrial volume index; LGE, late gadolinium enhancement; LV, left ventricle; LVEDVI, LV end-diastolic volume index; LVEF, LV ejection fraction; LVESVI, LV end-systolic volume index; LVMI, LV mass index; LVSVI, LV stroke volume index; MPG, mean pressure gradient; NYHA, New York Heart Association; PCI, percutaneous coronary intervention; PPG, peak pressure gradient; RVEDVI, right ventricular (RV) end-diastolic volume index; RVEF, RV ejection fraction; SAVR, surgical aortic valve replacement; SBP, systolic blood pressure; STS, society of thoracic surgeons; TAVR, transcatheter aortic valve replacement.

### Outcome

**Figure 2:**
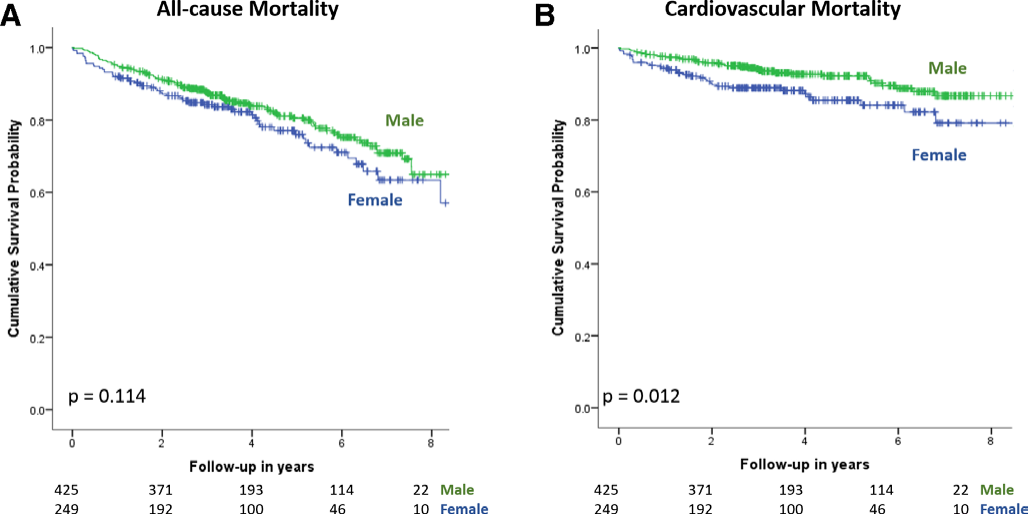
Kaplan-Meier survival curves for male and female patients: (a) all-cause mortality and (b) cardiovascular mortality.

**Figure 3:**
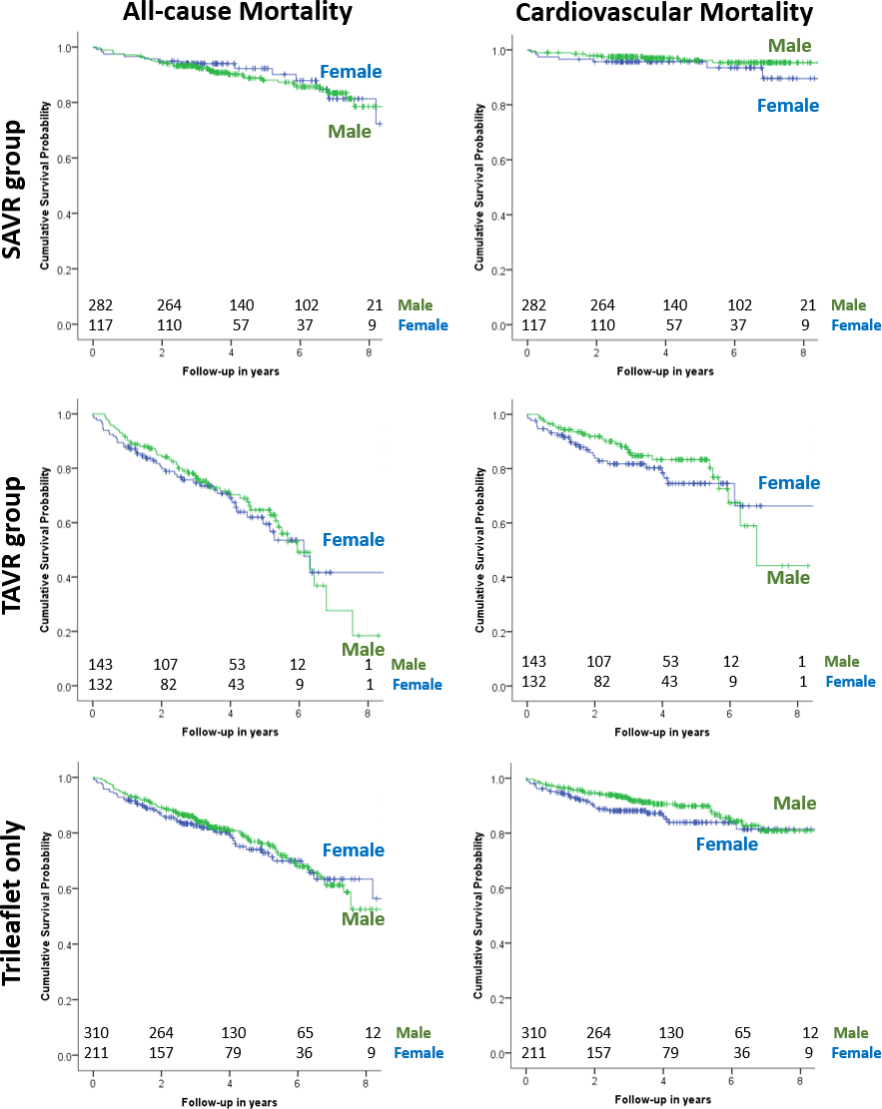
Kaplan-Meier survival curves for SAVR subgroup (top panel), TAVR subgroup (middle panel) and trileaflet aortic valve patients only (bottom panel), showing all-cause mortality (left panel) and cardiovascular mortality (right panel). All p>0.05 on Log-rank test. SAVR, surgical aortic valve replacement; TAVR, transcatheter aortic valve replacement.

During a median follow-up of 3.6 years (IQR 2.6–5.9 years, range 9.9 years), 145 (21.5%) patients died: 87 (20.5%) men and 58 (23.3%) women (table 2). There was no significant difference in all-cause mortality, but a higher incidence of cardiovascular mortality in women (13.7% vs 8.5%, p=0.012 on log-rank test) (figure 2). There were no significant sex differences in all-cause or cardiovascular mortality in the TAVR-only or SAVR-only subgroup (figure 3, table 2).

**Table 2:** Outcomes for all patients, SAVR group and TAVR group

Outcome	Male (n=425)	Female (n=249)	P value*
All patients: n (%)			
All-cause mortality	87 (20.5)	58 (23.3)	0.114
Cardiovascular mortality	36 (8.5)	34 (13.7)	**0.012**
SAVR group only: n (%)			
All-cause mortality	37 (13.1)	15 (12.8)	0.966
Cardiovascular mortality	11 (3.9)	8 (6.8)	0.206
TAVR group only: n (%)			
All-cause mortality	50 (35)	43 (32.6)	0.752
Cardiovascular mortality	25 (17.5)	26 (19.7)	0.418

*Log-rank test used.

SAVR, surgical aortic valve replacement; TAVR, transcatheter aortic valve replacement.

### Factors associated with outcome

As we have previously shown sex to be independently associated with cardiovascular mortality in the overall cohort,^17^ univariate associations were assessed with the inclusion of the variable’s interaction with sex in the regression model. Common associates of all-cause mortality (table 3) included age, atrial fibrillation, coronary disease, surgical risk scores, left atrial volume, LV end-systolic and stroke volumes, left and right ventricular EF (L/RVEF) and the presence/amount of LGE. In addition, BAV morphology and non-infarct pattern LGE were significant in men, while previous coronary intervention and infarct-pattern LGE were significant in women. AS severity was not associated in either sex.

**Table 3:** Univariate associations with all-cause mortality

	Male			Female			Interaction p value
Parameter	HR	95% CI	P value	HR	95% CI	P value	
Age	1.08	1.05 to 1.10	**<** **0.001**	1.07	1.04 to 1.10	**<** **0.001**	0.846
BMI	0.99	0.95 to 1.04	0.658	0.97	0.92 to 1.02	0.180	0.497
Hypertension	1.02	0.67 to 1.55	0.935	1.12	0.67 to 1.89	0.662	0.773
Diabetes	1.38	0.88 to 2.18	0.165	1.33	0.71 to 2.47	0.371	0.919
Atrial fibrillation	2.61	1.57 to 4.35	**<** **0.001**	1.91	1.03 to 3.54	**0.041**	0.442
BAV	0.22	0.11 to 0.45	**<** **0.001**	0.57	0.25 to 1.34	0.199	0.091
Previous MI	1.56	0.88 to 2.76	0.130	1.03	0.32 to 3.32	0.956	0.536
Previous PCI/CABG	1.13	0.67 to 1.90	0.654	2.83	1.57 to 5.11	**0.001**	**0.022**
Known CAD	1.58	1.03 to 2.42	**0.037**	2.61	1.48 to 4.62	**0.001**	0.165
ACE-I/ ARB	1.55	1.00 to 2.40	0.052	1.14	0.64 to 2.04	0.663	0.413
BB	1.08	0.70 to 1.67	0.717	1.40	0.82 to 2.37	0.215	0.467
Statin	1.10	0.70 to 1.73	0.692	1.30	0.75 to 2.27	0.352	0.638
STS score	1.21	1.13 to 1.29	**<** **0.001**	1.16	1.09 to 1.23	**<** **0.001**	0.343
EuroSCORE	1.08	1.03 to 1.13	**0.002**	1.12	1.07 to 1.18	**<** **0.001**	0.252
Echo data							
PPG	1.00	0.99 to 1.01	0.919	1.01	1.00 to 1.02	0.171	0.262
MPG	1.00	0.99 to 1.02	0.821	1.01	0.99 to 1.03	0.289	0.486
AVAI	0.25	0.02 to 2.62	0.247	0.37	0.03 to 4.70	0.443	0.824
CMR data							
LVEDVI	1.00	0.99 to 1.01	0.621	1.01	1.00 to 1.02	**0.049**	0.196
LVESVI	1.01	1.00 to 1.02	**0.028**	1.02	1.01 to 1.03	**<** **0.001**	0.066
LVSVI	0.97	0.95 to 0.99	**<** **0.001**	0.97	0.95 to 1.00	**0.026**	0.828
LVEF	0.98	0.97 to 0.99	**0.001**	0.97	0.95 to 0.98	**<** **0.001**	0.225
RVEDVI	1.00	0.98 to 1.01	0.487	1.00	0.99 to 1.02	0.696	0.450
RVEF	0.98	0.96 to 1.00	**0.012**	0.98	0.95 to 1.00	**0.027**	0.895
LAVI	1.01	1.00 to 1.02	**0.050**	1.01	1.00 to 1.03	**0.011**	0.548
LVMI	0.99	0.99 to 1.00	0.220	1.01	1.00 to 1.02	0.054	**0.024**
LV mass/volume	0.51	0.24 to 1.09	0.081	1.57	0.56 to 4.40	0.388	0.084
LGE presence	2.90	1.62 to 5.17	**<** **0.001**	2.05	1.16 to 3.62	**0.014**	0.402
LGE non-infarct	1.64	1.05 to 2.57	**0.030**	1.30	0.72 to 2.35	0.388	0.534
LGE infarct	1.57	0.97 to 2.54	0.067	2.69	1.34 to 5.43	**0.006**	0.213
LGE (g)	1.02	1.00 to 1.05	**0.022**	1.09	1.04 to 1.14	**0.001**	**0.026**
LGE (%)	1.06	1.02 to 1.11	**0.005**	1.12	1.06 to 1.19	**<** **0.001**	0.157

Abbreviations are as per table 1. Hazard ratios are per unit of the variable. Interaction p value is for interaction of variable with sex, which was included in the model.

The following variables were entered into a multivariate regression model in a single step: age, AF, BAV, CAD, LVEF, RVEF, LGE presence, LAVI and LVMI, as well as the sex-interaction variable with BAV and LVMI. The independently associated variables were age, LVEF and LGE in both sexes, with the addition of BAV in men (table 4). The same variables remained independent on entering all variables of interest and their sex-interaction variables into a backward selection model.

**Table 4:** Univariate associations with cardiovascular mortality

	Male (n=425)			Female (n=249)			Interaction p value
Parameter	HR	95% CI	P value	HR	95% CI	P value	
Age	1.09	1.05 to 1.14	**<** **0.001**	1.06	1.02 to 1.10	**0.003**	0.336
BMI	1.02	0.95 to 1.10	0.521	0.94	0.88 to 1.01	0.079	0.094
Hypertension	1.08	0.56 to 2.08	0.824	0.98	0.50 to 1.92	0.943	0.837
Diabetes	1.99	1.02 to 3.89	**0.044**	2.13	1.04 to 4.38	**0.039**	0.891
AF	4.05	1.99 to 8.23	**<** **0.001**	2.29	1.07 to 4.91	**0.033**	0.283
BAV	0.07	0.01 to 0.48	**0.007**	0.85	0.33 to 2.21	0.745	**0.023**
Previous MI	1.58	0.66 to 3.80	0.309	1.64	0.50 to 5.38	0.418	0.962
Previous PCI/CABG	1.03	0.45 to 2.36	0.945	3.56	1.73 to 7.32	**0.001**	**0.027**
Known CAD	1.76	0.91 to 3.40	0.092	3.15	1.55 to 6.40	**0.001**	0.237
ACE-I/ ARB	2.43	1.19 to 4.94	**0.014**	0.93	0.44 to 1.94	0.838	0.065
BB	1.61	0.84 to 3.11	0.152	1.31	0.66 to 2.60	0.440	0.666
Statin	1.66	0.78 to 3.53	0.190	1.07	0.53 to 2.16	0.844	0.407
STS score	1.27	1.17 to 1.39	**<** **0.001**	1.15	1.06 to 1.24	**<** **0.001**	0.080
EuroSCORE	1.11	1.04 to 1.18	**0.003**	1.13	1.07 to 1.20	**<** **0.001**	0.627
Echo data							
PPG	0.99	0.98 to 1.01	0.451	1.00	0.98 to 1.02	0.865	0.661
MPG	0.99	0.96 to 1.02	0.425	1.00	0.97 to 1.02	0.705	0.764
AVAI	0.04	0.00 to 1.73	0.092	1.34	0.06 to 27.67	0.851	0.148
CMR data							
LVEDVI	1.01	0.99 to 1.02	0.442	1.01	1.00 to 1.03	0.169	0.586
LVESVI	1.01	1.00 to 1.02	**0.014**	1.02	1.01 to 1.04	**0.006**	0.414
LVSVI	0.95	0.92 to 0.98	**<** **0.001**	0.97	0.93 to 1.00	0.050	0.423
LVEF	0.97	0.95 to 0.98	**<** **0.001**	0.96	0.94 to 0.98	**<** **0.001**	0.665
RVEDVI	1.01	0.99 to 1.03	0.443	1.00	0.98 to 1.02	0.751	0.446
RVEF	0.96	0.93 to 0.98	**0.001**	0.98	0.95 to 1.00	0.076	0.358
LAVI	1.02	1.01 to 1.03	**0.005**	1.02	1.00 to 1.03	**0.030**	0.673
LVMI	1.00	0.99 to 1.02	0.932	1.01	0.99 to 1.02	0.409	0.599
LV mass/volume	0.64	0.21 to 2.00	0.444	0.77	0.19 to 3.12	0.712	0.844
LGE presence	9.85	2.36 to 41.15	**0.002**	2.81	1.34 to 5.87	**0.006**	0.126
LGE non-infarct	1.67	0.85 to 3.31	0.139	1.29	0.62 to 2.70	0.494	0.614
LGE infarct	2.56	1.29 to 5.11	**0.008**	3.94	1.81 to 8.62	**0.001**	0.418
LGE (g) (FWHM)	1.03	1.00 to 1.06	**0.032**	1.10	1.04 to 1.17	**0.001**	**0.029**
LGE (%) (FWHM)	1.07	1.01 to 1.14	**0.019**	1.15	1.07 to 1.22	**<** **0.001**	0.151

Abbreviations are as per table 1.Hazard ratios are per unit of the variable. Interaction p value is for interaction of variable with sex, which was included in the model.

SAVR, surgical aortic valve replacement; TAVR, transcatheter aortic valve replacement.

Univariate associations with cardiovascular mortality (table 5)were similar, with the addition of diabetes for both sexes, lack of association of coronary disease and non-infarct pattern LGE in men, and fewer remodelling parameters in women. Independent associations with cardiovascular mortality included age and LGE in both sexes, when all variables shown in table 4 were entered into the model in a single step, with the addition of AF in men, and AF and diabetes in women on using a backward selection model.

**Table 5:** Table 5Multivariable associations with all-cause mortality and cardiovascular mortality

All-cause mortality					Cardiovascular mortality				
Parameter	HR	95% CI	P value	Interaction p value	Parameter	HR	95% CI	P value	Interaction p value
Age	1.06	1.04 to 1.09	**<** **0.001**		Age	1.07	1.03 to 1.11	**<** **0.001**	
AF	1.57	0.93 to 2.67	0.095		AF	1.89	0.96 to 3.70	0.065	
BAV (M) (F)	0.33	0.13 to 0.84	**0.020**	0.036	BAV (M) (F)	0.15	0.02 to 1.09	0.061	0.025
	1.35	0.50 to 3.67	0.554			2.32	0.82 to 6.61	0.114	
CAD	1.25	0.83 to 1.87	0.290		CAD	1.59	0.90 to 2.82	0.110	
LVEF	0.99	0.97 to 1.00	**0.044**		LVEF	0.99	0.97 to 1.00	0.077	
RVEF	1.00	0.98 to 1.02	0.812		LAVI	1.00	0.99 to 1.01	0.810	
LGE	2.27	1.47 to 3.51	**<** **0.001**		LGE	3.17	1.65 to 6.09	**0.001**	
LAVI	1.00	0.99 to 1.01	0.316		Diabetes	1.66	0.93 to 2.97	0.086	
LVMI (M) (F)	0.99	0.98 to 1.00	0.191	0.030					
	1.01	1.00 to 1.03	0.108						

Multivariate analysis performed with all independent variables entered into the model in one step. Interaction p value is shown for those variables which had interaction with sex on univariate analysis, and had their sex-interaction variable included in the multivariate model, for which separate HR(CI) are shown for men and women. The HR (CI) are the same for both sexes for the other variables. Abbreviations are as per table 1. ‘LGE’ implies ‘LGE presence’ as a categorical variable. On testing just STS score and LGE, both remain independent on forward stepwise selection.

We also performed multivariable analysis with stepwise selection of LGE in addition to STS score, and both remained independently associated with all-cause and cardiovascular mortality in both sexes, with LGE providing incremental prognostic information.

### Exclusion of BAV patients

We excluded BAV to remove the bias of the younger BAV subgroup in men (mean age 63.3±11.4 vs 75.6±8.8 years, p<0.01 and lower incidence of diabetes, AF and coronary disease). This left 313 male and 212 female patients, with similar sex differences in remodelling parameters (higher mass, volumes and mass/volume in men), but differences in LVEF, RVEF and LV stroke volume index no longer being significant (online supplementary table 1). There was no significant sex difference in all-cause or cardiovascular mortality (table 2, figure 2, online supplementary table 2). Univariate associations, corrected for sex-interactions, are presented in online supplementary tables 3,4. On multivariable analysis entering all variables in a single step, independent associations of all-cause mortality were age and LGE in both sexes, with the addition of AF in women when using a backward selection model. Independent associations of cardiovascular mortality were age, AF and LGE in men, with the addition of diabetes in women. The same variables remained significant on backward stepwise selection (online supplementary table 5).

10.1136/heartjnl-2019-314987.supp1 Supplementary data

## Discussion

This large multicentre CMR study confirms sex differences in LV remodelling. Although there was no significant difference in all-cause mortality, cardiovascular mortality was higher in women. However, these observed differences are accounted for by men being younger with more BAV, while women having more TAVR likely due to higher risk profile, possibly reflecting differences in the referral practices for male and female patients. LGE was independently associated with all-cause and cardiovascular mortality in both sexes.

### Sex differences in LV remodelling

We have confirmed findings from previous single-centre studies utilising CMR^3–5^ that for a similar severity of AS, men demonstrate more advanced LV remodelling, with larger indexed volumes, mass, mass/volume and lower EF, in addition to more focal fibrosis (LGE). While women were thought to demonstrate more concentric remodelling based on older TTE measurements of higher relative wall thickness,^23 24^ this was traditionally based on a single basal slice, often using M-mode, which has many assumptions about the shape and symmetry of the LV. CMR overcomes many of these limitations and is now regarded as the gold standard for quantitative LV assessment, and the finding of greater concentric remodelling (ie, higher mass/volume) in men has now been confirmed in other CMR studies.^4^ Despite this seemingly more maladaptive response in men, female sex has been associated with both earlier symptom onset^16^ and worse mortality in AS.^9–11^ Putative mechanisms for these differences include higher wall stress in women due to less adaptive concentric remodelling for a similar degree of pressure overload, which may contribute to earlier symptoms. In fact, in this study, a greater proportion of women had New York Heart Association (NYHA) III–IV symptoms than men.

### Referral patterns for intervention

There were more men in the SAVR group (71%), a difference that persisted even after removing those with BAVs (69%), with the proportions being almost equal for TAVR. This discrepancy in referral for surgical intervention has been noted in both historical single-centre studies (68% in a retrospective analysis of consecutive procedures^10^) and a multicentre analysis of an American national database (63%).^25^ This is also confirmed by the UK national cardiac surgery database reporting 60% of patients undergoing AVR being men, rising to around 70% for AVR and coronary artery bypass graft.^26^ On the contrary, the proportions are almost equal for TAVR in both single-centre studies^14^ and larger registries.^27^ Both a disparity in referral for testing and referral for surgery have been suggested,^25 28^ possibly due to greater incidence of comorbidities or risk scores at presentation in female participants.^10 11 27^ Other data suggest a greater benefit of TAVR in high-risk female patients,^15 27^ possibly leading to more women being referred for TAVR. There is also the possibility of perceived and/or real higher incidence of patient-prosthesis mismatch in women leading to a bias towards TAVR referral, and higher cardiovascular mortality. The female patients in our study were indeed older, more symptomatic (greater proportion of NYHA III–IV symptoms) and had higher surgical risk scores at the time of intervention.

### Factors associated with outcome

Age and LGE were the common factors associated independently with all-cause and cardiovascular mortality in both sexes, with LVEF also significant for all-cause mortality. LGE also provided incremental prognostic information to STS score, which incorporates many clinical variables. The fact LVEF was not associated with cardiovascular outcomes may relate to the fact that few patients had impaired LV function. As expected, there was a greater proportion of BAVs in the male subgroup. BAV is thought to represent a different pathology to degenerative trileaflet AS, with better survival post AVR in a large age and sex matched cohort.^29^ Despite its exclusion, age and LGE remained significant for all-cause mortality, where as LGE was significant for cardiovascular mortality in only men, with the addition of diabetes in women and AF in both sexes, as the younger and fitter BAV patients were excluded.

### Is earlier intervention warranted?

Given that women in our multicentre study were older, more symptomatic, with higher risk scores, and more frequently referred for TAVR, differences in care pathways for male and female patients may exist. It is possible that due to more advanced remodelling, men tend to get referred earlier for a similar degree of AS. In addition, subjective differences in interpretation and acknowledgement of symptoms may add to this potential referral bias. The association of LGE with poor outcome even after intervention in both sexes, supports the need for earlier intervention in AS, before fibrosis develops, and the need for trials to establish the best stratification tools. The EVOLVED (Early Valve Replacement guided by Biomarkers of Left Ventricular Decompensation in Asymptomatic Patients with Severe AS) trial is underway, which specifically addresses this question (NCT03094143).

There are limitations to our study.^17^ This was an observational study of patients at surgical centres with an interest in CMR, potentially introducing selection bias. Certain patient groups with a contraindication to CMR were excluded (advanced renal failure and non-compatible devices). LGE was not performed in a small minority of patients (n=61), and T1 mapping and biomarker analysis are also lacking. We did not objectively measure frailty or exercise capacity. The prevalence of occult amyloidosis, which has been shown to be present in 4%–8% of severe AS patients,^30^ is also unknown, as biopsy or radiolabelled scans were not performed as part of this study.

## Conclusion

Men demonstrate more advanced remodelling in response to a similar degree of AS. The higher cardiovascular mortality observed in women following AVR is accounted for by women having less BAV and higher risk scores resulting in more TAVR. LGE is associated with a worse prognosis in both sexes.

**Key questions**

**What is already known on this subject?**

- Sex differences in remodelling and post-aortic valve replacement (AVR) outcome have been reported in aortic stenosis (AS), with men demonstrating more advanced remodelling and worse outcomes in women in some studies.

**What might this study add?**

- In this large multicentre, prospective longitudinal outcome study of patients undergoing cardiovascular magnetic resonance imaging before surgical/transcatheter AVR (SAVR/TAVR), during a median follow-up of 3.6 years (IQR 2.6–5.9 years), there was no significant difference in all-cause mortality (23.3% vs 20.5%, p=0.114), but higher cardiovascular mortality in women (13.7% vs 8.5%, p=0.012). However, this finding did not persist after accounting for type of intervention and prevalence of bicuspid aortic valve. A greater proportion of women underwent TAVR (53% vs 33.6%, p<0.001), with higher risk scores. Late gadolinium enhancement presence was associated with adverse prognosis in both sexes, even after AVR.

**How might this impact on clinical practice?**

- Clinicians need to be aware of a possible bias in not referring female patients with severe AS for intervention and that when other risk factors are accounted for, cardiovascular and all-cause mortality are similar in men and women. Further studies are needed to define the best stratification tools in AS, with possible sex-specific cut-offs to define severity and timing of intervention.
