# Supplementary material for: Sex differences in left ventricular remodelling, myocardial fibrosis and mortality after aortic valve replacement
Source: Heart. 2019 Aug 29;105(23):1818–24. doi: 10.1136/heartjnl-2019-314987 (PMC6900227; doi:10.1136/heartjnl-2019-314987)
Supplement: Supplementary data [file heartjnl-2019-314987supp001.pdf]

Supplemental tables for:

Sex differences in outcome following AVR: Data from British Society of Cardiovascular Magnetic Resonance Valve Consortium

Supplemental Table-1. Baseline characteristics for male and female patients after excluding BAV patients

Variable	Male (n= 313)	Female (n= 212)	p-value
Age (years)	74.9 ± 8.3	76.8 ± 9.2	**0.015**
BMI (kg/m^2)	27.9 ± 4.7	27.2 ± 5.8	0.192
BSA (cm^2)	1.97 ± 0.19	1.74 ± 0.20	**<0.001**
SBP (mmHg)	135.1 ± 20.0	137.4 ± 22.2	0.357
Hypertension (n (%))	173 (55.3)	111 (52.4)	0.511
AF (n (%))	42 (13.4)	32 (15.1)	0.588
Diabetes (n (%))	82 (26.2)	43 (20.3)	0.118
Known CAD (n(%))	125 (39.9)	45 (21.2)	**<0.001**
Previous PCI (n(%))	28 (9.3)	25 (12.0)	0.340
Previous CABG (n(%))	44 (14.7)	12 (5.7)	**0.002**
ACEI/ARB (n (%))	139 (47.0)	77 (39.7)	0.113
Beta-blocker (n (%))	117 (37.6)	81 (38.2)	0.892
Aldosterone antagonist (n (%))	16 (5.1)	14 (6.6)	0.481
Statin (n (%))	208 (67.5)	124 (59.3)	0.056
STS Mortality Score (%)	1.82 [1.22, 2.80]	2.60 [1.56, 4.49]	**<0.001**
EuroSCORE II (%)	1.86 [1.09, 3.66]	2.44 [1.53, 4.48]	**0.001**
**NYHA Class**			
I	47 (16.5)	14 (7.4)	**0.007**
II	111 (39.1)	68 (35.8)	
III	117 (41.2)	97 (51.1)	
IV	9 (3.2)	11 (5.8)	
**Echocardiographic data**			

MPG (mmHg)	47.2 ± 13.5	49.1 ± 14.8	0.174
PPG (mmHg)	80.0 ± 21.1	83.8 ± 23.3	0.080
AVAI (cm^2/m^2)	0.38 ± 0.12	0.38 ± 0.13	0.854
**Cardiovascular magnetic resonance data**			
LVEDVI (ml/m^2)	86.4 ± 26.5	79.8 ± 22.9	**0.004**
LVESVI (ml/m^2)	39.3 ± 24.8	34.5 ± 20.8	**0.020**
LVSVI (ml/m^2)	47.1 ± 12.0	45.3 ± 10.4	0.091
LVEF (%)	60.0 [49.0, 68.0]	61.0 [52.0, 69.0]	0.130
LVMI (g/m^2)	87.0 ± 23.7	75.3 ± 21.6	**<0.001**
LV Mass/Volume	1.05 ± 0.29	0.97 ± 0.26	**0.002**
RVEDVI (ml/m^2)	72.1 ± 16.9	66.2 ± 17.2	**<0.001**
RVEF (%)	64.0 [57.0, 70.0]	65.0 [59.0, 73.0]	0.073
LAVI (ml/m^2)	57.3 ± 21.5	59.1 ± 21.6	0.362
LGE present (n(%))	183 (62.0)	78 (42.2)	**<0.001**
Non-infarct pattern (n(%))	110 (37.3)	51 (27.6)	**0.028**
Infarct-pattern (n(%))	73 (24.7)	27 (14.6)	**0.008**
LGE mass (g) (FWHM)	1.93 [0.00, 6.56]	0.00 [0.00, 2.49]	**<0.001**
% LGE (%) (FWHM)	1.21 [0.00, 3.61]	0.00 [0.00, 1.91]	**<0.001**

Abbreviations: BMI:body mass index, BSA:body surface area, SBP:systolic blood pressure, AF:atrial fibrillation, CAD:coronary artery disease, PCI:percutaneous coronary intervention, CABG:coronary artery bypass graft, ACE-I:angiotensin-converting enzyme inhibitor, ARB:angiotensin II receptor blocker, BAV:bicuspid aortic valve, STS:Society of Thoracic Surgeons, MPG:mean pressure gradient, PPG:peak pressure gradient, AVAI:aortic valve area index (to BSA), LV:left ventricle, LVEDVI:LV end-diastolic volume index, LVESVI:LV end-systolic volume index, LVSVI:LV stroke volume index, LVEF:LV ejection fraction, LVMI:LV mass index, RVEDVI:right ventricular(RV)end-diastolic volume index, RVEF:RV ejection fraction, LAVI:left atrial volume index, LGE:late gadolinium enhancement.

Supplemental Table-2. Intervention type and outcome in patients after excluding BAV patients

	Male (n= 313)	Female (n= 212)	p-value*
**Outcome**			
All-cause mortality (n(%))	79 (25.2)	52 (24.5)	0.766
CVS mortality (n(%))	35 (11.2)	29 (13.7)	0.226
**Intervention type**			
SAVR (n(%))	182 (58.1)	83 (39.2)	**<0.001**
TAVR (n(%))	131 (41.9)	129 (60.8)	**<0.001**

*Log-rank test used for comparing mortality between sexes, chi-square test used for type of intervention.

Abbreviations: CVS: cardiovascular, S/TAVR: surgical/ transcatheter aortic valve replacement.

Supplemental Table-3. Univariate predictors of all-cause mortality in male and female patients after excluding BAV patients

	MALE			FEMALE			Interaction p-value
**Parameter**	**HR**	**95% CI**	**p-value**	**HR**	**95% CI**	**p-value**	
**Age**	1.07	1.05 – 1.10	**<0.001**	1.07	1.05 – 1.10	**<0.001**	0.717
**BMI**	0.97	0.94 – 1.01	0.136	0.97	0.94 – 1.01	0.151	0.932
**Hypertension**	0.86	0.58 – 1.27	0.434	1.06	0.67 – 1.67	0.820	0.404
**Diabetes**	1.14	0.73 – 1.77	0.568	1.46	0.82 – 2.62	0.200	0.465
**Atrial Fibrillation**	2.27	1.39 – 3.72	**0.001**	2.00	1.10 – 3.65	**0.023**	0.732
**Previous MI**	1.33	0.76 – 2.31	0.322	1.17	0.37 – 3.71	0.785	0.849
**Previous PCI/CABG**	0.97	0.58 – 1.61	0.898	2.54	1.47 – 4.39	**0.001**	**0.006**
**Known CAD**	1.24	0.83 – 1.85	0.289	2.40	1.42 – 4.08	**0.001**	**0.026**
**ACE-I/ ARB**	1.56	1.04 – 2.34	**0.031**	1.19	0.69 – 2.05	0.535	0.345
**BB**	1.03	0.68 – 1.57	0.877	1.32	0.82 – 2.12	0.254	0.384
**Statin**	0.96	0.64 – 1.45	0.862	1.16	0.74 – 1.83	0.519	0.398
**STS score**	1.17	1.10 – 1.24	**<0.001**	1.16	1.10 – 1.22	**<0.001**	0.827
**EURO Score**	1.05	1.01 – 1.10	**0.025**	1.11	1.06 – 1.16	**<0.001**	0.071
Echo data							
**PPG**	1.00	0.99 – 1.01	0.962	1.00	0.99 – 1.01	0.678	0.482
**MPG**	1.01	0.99 – 1.02	0.478	1.01	0.99 – 1.02	0.362	0.732
**AVAI**	0.38	0.06 – 2.39	0.303	0.51	0.08 – 3.29	0.482	0.595
CMR data							
**LVEDVI**	1.01	1.00 – 1.01	0.086	1.01	1.00 – 1.02	**0.043**	0.337
**LVESVI**	1.01	1.00 – 1.02	**0.001**	1.02	1.01 – 1.03	**<0.001**	0.056
**LVSVI**	0.97	0.95 – 0.99	**<0.001**	0.97	0.95 – 0.99	**<0.001**	0.827
**LVEF**	0.97	0.96 – 0.99	**<0.001**	0.98	0.97 – 0.99	**<0.001**	0.698
**RVEDVI**	1.00	0.99 – 1.01	0.968	1.00	0.99 – 1.01	0.756	0.564
**RVEF**	0.97	0.96 – 0.99	**<0.001**	0.97	0.96 – 0.99	**<0.001**	0.741

**LAVI**	1.01	1.00 – 1.02	**0.012**	1.01	1.00 – 1.02	**0.005**	0.514
**LVMI**	1.00	0.99 – 1.01	0.863	1.00	0.99 – 1.01	0.546	0.374
**LV Mass/Volume**	0.66	0.36 – 1.22	0.181	0.70	0.35 – 1.39	0.303	0.758
**LGE presence**	2.36	1.53 – 3.63	**<0.001**	2.76	1.62 – 4.70	**<0.001**	0.515
**LGE non-infarct**	1.77	1.18 – 2.67	**0.006**	1.70	0.97 – 2.99	0.064	0.894
**LGE infarct**	1.36	0.86 – 2.16	0.192	2.29	1.14 – 4.57	**0.019**	0.191
**LGE (g)**	1.05	1.02 – 1.08	**<0.001**	1.09	1.04 – 1.14	**<0.001**	0.140
**LGE %**	1.08	1.04 – 1.13	**<0.001**	1.13	1.06 – 1.19	**<0.001**	0.235

Abbreviations as in Table-1. Hazard ratios are per unit of the variable. Interaction p-value is for interaction of variable with sex, which was included in the model.

Supplemental Table-4. Univariate predictors of cardiovascular mortality in male and female patients excluding BAV patients

	MALE			FEMALE			Interaction p-value
**Parameter**	**HR**	**95% CI**	**p-value**	**HR**	**95% CI**	**p-value**	
**Age**	1.07	1.04 – 1.11	**<0.001**	1.07	1.04 – 1.11	**<0.001**	0.508
**BMI**	0.98	0.93 – 1.03	0.339	0.98	0.93 – 1.03	0.492	0.504
**Hypertension**	0.81	0.46 – 1.44	0.473	1.11	0.59 – 2.09	0.745	0.374
**Diabetes**	1.41	0.75 – 2.63	0.288	2.86	1.46 – 5.58	**0.002**	0.085
**AF**	2.85	1.47 – 5.51	**0.002**	2.88	1.36 – 6.11	**0.006**	0.982
**Previous MI**	1.15	0.49 – 2.68	0.745	2.19	0.68 – 7.04	0.190	0.365
**Previous PCI/CABG**	0.80	0.36 – 1.78	0.586	3.60	1.86 – 6.97	**<0.001**	**0.002**
**Known CAD**	1.13	0.62 – 2.06	0.691	3.38	1.75 – 6.51	**<0.001**	**0.005**
**ACE-I/ ARB**	1.55	0.89 – 2.75	0.131	1.40	0.68 – 2.90	0.363	0.789
**BB**	1.16	0.64 – 2.10	0.618	1.50	0.78 – 2.89	0.224	0.503
**Statin**	1.17	0.64 – 2.12	0.610	1.43	0.74 – 2.75	0.284	0.507
**STS score**	1.19	1.09 – 1.29	**<0.001**	1.18	1.11 – 1.26	**<0.001**	0.944
**EURO Score**	1.06	0.99 – 1.13	0.084	1.13	1.08 – 1.19	**<0.001**	0.055
Echo data							
**PPG**	0.99	0.98 – 1.01	0.189	1.00	0.98 – 1.01	0.456	0.223
**MPG**	0.98	0.96 – 1.01	0.190	0.99	0.97 – 1.01	0.389	0.367
**AVAI**	0.32	0.02 – 4.62	0.405	0.92	0.07 – 11.49	0.949	0.189
CMR data							
**LVEDVI**	1.01	1.00 – 1.02	0.320	1.01	1.00 – 1.02	0.081	0.121
**LVESVI**	1.01	1.00 – 1.02	**0.016**	1.02	1.01 – 1.03	**<0.001**	**0.029**
**LVSVI**	0.95	0.93 – 0.98	**<0.001**	0.96	0.94 – 0.98	**0.001**	0.235
**LVEF**	0.96	0.95 – 0.98	**<0.001**	0.97	0.95 – 0.98	**<0.001**	0.206
**RVEDVI**	1.00	0.99 – 1.01	0.909	1.00	0.99 – 1.02	0.691	0.288
**RVEF**	0.96	0.94 – 0.98	**<0.001**	0.96	0.95 – 0.98	**<0.001**	0.186
**LAVI**	1.01	1.00 – 1.03	**0.020**	1.02	1.01 – 1.03	**0.002**	0.273

**LVMI**	1.00	0.99 – 1.01	0.843	1.00	0.99 – 1.02	0.682	0.254
**LV Mass/Volume**	0.53	0.21 – 1.33	0.175	0.63	0.23 – 1.73	0.369	0.482
**LGE presence**	3.42	1.72 – 6.83	**<0.001**	5.39	2.52 – 11.52	**<0.001**	0.137
**LGE non-infarct**	1.62	0.90 – 2.92	0.107	1.96	0.94 – 4.12	0.074	0.645
**LGE infarct**	1.76	0.93 – 3.30	0.081	4.25	1.97 – 9.19	**<0.001**	0.051
**LGE g (FWHM)**	1.05	1.01 – 1.09	**0.008**	1.12	1.06 – 1.18	**<0.001**	**0.024**
**LGE % (FWHM)**	1.08	1.02 – 1.15	**0.015**	1.17	1.10 – 1.24	**<0.001**	**0.048**

Abbreviations as in Table-1. Hazard ratios are per unit of the variable. Interaction p-value is for interaction of variable with sex, which was included in the model.

Supplemental Table-5. Multivariable associations with All-cause mortality and Cardiovascular mortality in male and female patients after excluding BAV patients

**ALL-CAUSE MORTALITY**					**CARDIOVASCULAR MORTALITY**				
**Parameter**	**HR**	**95% CI**	**p-value**	**Interaction p-value**	**Parameter**	**HR**	**95% CI**	**p-value**	**Interaction p-value**
**Age**	1.07	1.04 – 1.10	**<0.001**		**Age**	1.08	1.04 – 1.12	**<0.001**	
**AF**	1.61	0.94 – 2.76	0.085		**AF**	2.24	1.14 – 4.43	**0.020**	
**CAD (M)**	1.10	0.67 – 1.81	0.696		**Diabetes (M)**	1.22	0.53 – 2.82	0.642	
**(F)**	1.55	0.77 – 3.13	0.221	0.438	**(F)**	2.98	1.24 – 7.17	**0.015**	0.144
**LVEF**	0.99	0.98 – 1.00	0.121		**CAD (M)**	1.08	0.50 – 2.32	0.840	
**RVEF**	1.00	0.98 – 1.02	0.736		**(F)**	1.94	0.82 – 4.60	0.134	0.321
**LGE**	2.45	1.55 – 3.86	**<0.001**		**LVEF**	0.99	0.97 – 1.01	0.236	
**LAVI**	0.99	0.98 – 1.00	0.138		**RVEF**	0.99	0.97 – 1.02	0.628	
					**LGE**	3.32	1.64 – 6.73	**0.001**	
					**LAVI**	0.99	0.98 – 1.01	0.366	

Multivariate analysis performed with all independent variables were entered into the model in one step. Interaction p-value is shown for those variables which had interaction with sex on univariate analysis, and the interaction term included in the multivariate model, for which separate HR(CI) for male and female are shown. The HR (CI) are the same for both sexes for the other variables. Abbreviations as per Table-1. ‘LGE’ implies LGE presence as a categorical variable. On testing just STS score and LGE, both remain independent on forward stepwise selection. Hazard ratios are per unit of the variable.
